# MESS to live with schizophrenic parental history: A systematic review of developmental checkpoints

**DOI:** 10.1371/journal.pone.0313531

**Published:** 2025-01-15

**Authors:** Maryam Khan, Rabia Batool, Uzma Mushtaq, Shakir Iqbal, Sana Shaheen, Aimen Zafar Butt, Anees Ahmed

**Affiliations:** 1 SWPS University, Wroclaw, Poland; 2 Department of Psychology, Muslim Youth University, Islamabad, Pakistan; 3 Department of Psychology, Capital University of Science and Technology, Islamabad, Pakistan; 4 Department of Professional Psychology, Bahria University, Islamabad, Pakistan; Jawaharlal Institute of Postgraduate Medical Education and Research, INDIA

## Abstract

Parental history of schizophrenia, a complex and multifaceted psychological disorder, is recognized as a well-established risk factor in the development of the disorder among offspring. However, the developmental patterns of such children and adolescents before the onset of the problem have not yet been systematically documented. We present a comprehensive account of developmental checkpoints essential for preventing it from occurring. This review embarks on a detailed explanation of the domains requiring serious attention during the development of an individual with such a familial history. We examined a diversified set of studies comparing the developmental patterns of children with or without (a comparative) a parental history of schizophrenia and highlighted the areas of concern for the later development of the problem among the first group. We included the peer-reviewed articles, published in English based on children and adolescents, found in Web of Science, PubMed, and PsychInfo databases and separate citation searches. We summarized our findings under MESS typology covering motor development, emotional and behavioral issues, speech and hearing impairments, and socio-cognitive aspects as essential features of a child’s development serving as a guide to prevent the onset of psychological complications.

## Introduction

Parental history of schizophrenia, a complex and multifaceted psychological disorder, is recognized as a well-established risk factor in the development of the disorder among offspring. In line with this thought, most of the scientific exploration documented it as an etiological facet of ongoing disorder. However, in recent times, a variety of risk factors from multiple domains like familial background, environmental exposure, and psychological vulnerabilities, have been identified. Despite all that, there is consistent evidence that a child with a parental history of schizophrenia experiences an increased risk of developing the problem as exhibited by familial high-risk studies [1].

Literature presents high-risk (HR) studies investigating the etiological factors for those who are at risk to develop a specific disorder. Such research presents that genetic hereditary is the most significant risk factor in the development of schizophrenia. As there is little evidence for the other environmental factors, only established method for the identification of vulnerable children is to look up for the positive family history. The risk of development of schizophrenia is approx. 10% among the children whose one of the parent had the disorder and this increases to 50% if both parents were affected compared to the 1% risk of this disorder in the general population [2].

Further, the research covering the follow-up studies with the children who developed this disorder in their childhood began in 1940s. These studies reported a deviated neurological maturation among such children. Likewise, a condition called neuro-integrative defect also called pandysmaturation (PDM), is based on transient retardation in terms of motor or visual motor development of a child. It further included retarded skeletal growth and abnormal profile of functioning. The researchers tested this in an infant study conducted in New York with 7 out of 12 high risk infant and 1 out of 12 control group infants had PDM. Later in life, all those seven infants developed schizophrenia or related disorder. Thus, most of studies started investigating early childhood functioning mainly neurological and motor development of children [3].

Additionally, a review of genome-wide association research and copy number variation explorations demonstrated that the picture is not this much clear and transparent. Also, the pure genetic effect for schizophrenia was not even guaranteed by high hereditary estimates. Given the fact that children with a parental history of psychosis are likely to experience the familial environment or exposure that has been the cause of the psychotic disorder, for instance, parental maladaptive communication practices or treatment in early childhood [3]. Thus, to assert that it might be the gene-environment interaction hypothesis operating in such dynamics rather than just genetic predisposition [4].

Eventually, concerning the socio-environmental factors, clinical examinations [5], prospective explorations [6] and studies based on the general population [7] have identified childhood complications like exposure to trauma or traumatic treatment or related interpersonal negligence associated with schizophrenia. Most of the work was focused on sexual abuse and psychosis [8] but disrupted familial set-ups like parental separation or death significantly increased the chances of psychosis in comparison to other risk factors [9].

As we attempt to present a comprehensive picture of developmental patterns, we found another trend in schizophrenia-based research. Apart from genetic and environmental factors, there has been some developmental comparison between children with or without a parental history of schizophrenia. These comparisons not only highlighted the differences but also presented the potential outcomes reported in the development of such children. Therefore, we tried to incorporate literature addressing the personal developmental aspects of a child, other than environmental factors only. Research in this context suggests cognitive impairments as the established indicator of schizophrenic vulnerability [10]. Neurological deficits, motor development issues [11], speech anomalies [12], hearing impairments [13], and poor school performance [14] at the age of 16 years and later cognitive functioning in schizophrenia correlated with motor developmental delays [15].

Lastly, along with the information presented above, we consider that most of the work (even systematic reviews) is designated to the understanding of the problem after the onset of schizophrenia [16]. But, we are focusing on the issue before the onset or just with the potential risk of development of the problem. Thus, the attempt is to provide a simple blueprint for parents or the common public rearing a child with a parental history of schizophrenia. We intend to directly find the developmental checkpoints associated with the development of schizophrenia later in life, so those areas are to be monitored carefully and also, to present some avenues to look up for if any discrepancy or deviation is observed in the early development of child.

We targeted the three streams i.e., history of parental illness and risk of developing a problem, domains of struggle—requiring cautions or vigilance by the caregiver—for a child, and possible prevention measures. Consequently, we conducted a specified systematic review incorporating the studies on schizophrenia, importantly covering the element of prevention, spanning at least 20 years.

## Material and methods

### Search strategy and selection criteria

We conducted a systematic literature search based on PRISMA guidelines [17], in October–November 2023. We considered the studies on schizophrenia, prevention, and intervention from the last 20 years or more and began our search with a general Google search, PubMed, PsycINFO, and Web of Science using the following search string:

((schizophren*) OR (schizoaffective) OR (psychosis) OR (psychoses) OR (psychotic)) AND ((longitudinal) OR (“long-term”) OR (follow-up) or (“follow-up”)) AND ((“20 year*”) OR (“20-year*”) OR (“twenty year*”) OR (“twenty-year*”) OR (“very long term”) OR (“very long-term”)) AND ((child) OR (adolescent)) AND ((symptoms) OR (risk)) AND ((prevention) OR (intervention)) AND ((parental history) OR (familial history) OR (maternal history))

Finally, reference lists of the relevant systematic reviews and articles were screened to consider the additional papers covering the desired information. The study has the following inclusion and exclusion criteria:

1. Peer-reviewed studies on schizophrenia and prevention, in the English language containing indicators of the potential areas of risk, and caution in the development of the problem due to parental history of schizophrenia.
2. Studies from at least the last 20 years.
3. The study must provide information on the prevention of the problem or the potential indicator of developing schizophrenia.
4. Articles containing information on schizophrenia, schizoaffective disorder, schizophreniform disorder, or non-affective psychosis were considered to be included but the studies having no differentiation between types of psychosis, e.g., FEP cohorts, were decided to be excluded.
5. The sample was decided to be children and adolescents only.

### Study selection and retrieval

After the decision on inclusion and exclusion criteria with the whole team, two independent authors screened the titles and abstracts of the studies, after the removal of duplicates. The reference lists of the included articles were also screened to find the relevant studies. Then a full-text review was also carried out by two independent authors, a consensus was built with crosschecking by all authors collectively, and the points of conflict were discussed with the senior faculty members and resolved carefully.

### Quality assessment

The quality of the studies or potential risk of bias was assessed using 7 items based on the National Institutes for Health (NIH) Study Quality Assessment Tool for Systematic Reviews and Meta-Analyses [18]. We included the relevant questions from the mentioned protocols. These covered the adequately formulated question, specified inclusion-exclusion criteria, risk of selection bias, measurement bias, and publication bias. Two independent reviewers rated the studies according to the quality check and based on the scores (the higher the score, the higher the quality and the lower the risk of bias), the studies were categorized and reported as good, fair, or poor(the details of the quality assessment tool and the scores for each study are available).

### Data extraction

We extracted the desired data and placed them into an Excel spreadsheet, version LTSC MSO (16.0.14332.20587) 64-bit. It included the first authors’ contact details, year of publication, location of the study, study design, sample, sample size, gender, age, symptoms, risk factors, areas of prevention, and measures to build resilience or prevent the problem from developing. We extracted clinical, social, and overall risk factors from the studies. We also considered the parental history of schizophrenia and the details about the key challenges in development as compared to those having no familial history of the problem. The major findings were extracted and addressed according to the three streams of information we are focusing on. The data was either extracted from the texts, tables, or from the figures available in the selected studies. Conflicts were resolved by mutual discussion and consensus was built in the specific cases among the team members. The complete information about the extracted data and related aspects is available in Table 1.

**Table 1:** Summary of the included studies (*N* = 14).

S#	Title	Authors/Year	Design/Method	Sample	Measures	Findings
1.	Interaction between parental psychosis and early motor development and the risk of schizophrenia in a general population birth cohort	Keskinen et al., 2015 [11]	A prospective study	N = 10,283	Regular visits to Finnish child welfare clinics.	Findings suggested that delayed motor development in infancy and parental psychosis was associated with increasing the risk of schizophrenia.
2.	Social, familial and psychological risk factors for psychosis: A birth cohort study using the Danish Registry System	Shevlin et al., 2016 [7]	A prospective study	N = 54,458	Danish Civil Registration System and the Danish Psychiatric Central Register.	The findings indicate that familial, environmental, and psychological elements contribute significantly to the development of psychotic disorder.
3.	Polygenic Risk Score, Parental Socioeconomic Status, Family History of Psychiatric Disorders, and the Risk for Schizophrenia A Danish Population-Based Study and Meta-analysis	Agerbo et al., 2015 [26]	A prospective study	N = 866	Neonatal biobanks and national registers.	The results showed that polygenic risk score, family psychiatric history, and socioeconomic status have an association with schizophrenia. Further, the family history of schizophrenia/psychoses is partly mediated through the individual’s genetic liability.
4.	Executive functions in 7 year old children of parents with schizophrenia or bipolar disorder compared with controls: The Danish High Risk and Resilience Study—VIA 7, a population based cohort study	Spang et al., 2021 [28]	A prospective study	N = 522	Behavior Rating Inventory of Executive Function (BRIEF)	Study findings revealed that the children with familial high risk of schizophrenia displayed significant impairments in executive functioning. In addition to this, they had an odds ratio of 3.7 (2.0–6.9) of having clinically significant global Executive Functioning impairments compared to controls in everyday-life situations.
5.	Characterizing Speech Heterogeneity in Schizophrenia-Spectrum Disorders	Oomen et al., 2022 [25]	Hierarchical clustering method	N = 289	1. Open-ended, semi-structured interviews. 2. Positive and Negative Syndrome Scale. 2. Brief Assessment of Cognition for Schizophrenia.	The research results showed that the patients with Schizophrenia Spectrum Disorders (SSD) have significantly more interrupted speech, longer pause duration, lower pitch variability, and a lower proportion of time articulating compared to healthy controls.
6.	Influence of Family and Childhood Memories in the Development and Manifestation of Paranoid Ideation	Carvalho et al., 2015 [22]	Cross-sectional study	N = 187	1. General Paranoia Scale (GPS) 2. Paranoia Checklist (PC) Beck’s Depressive Inventory (BDI) 3. Early Life Experiences Scale (ELES) 4. Childhood Experiences of Care and Abuse Questionnaire (CECA-Q) 5. The Antipathy and Neglect Scales. 6. Bully/Victim Questionnaire (BVQ)	Results suggested that memories of parental behaviours characterized by antipathy from parents, submissiveness and bullying victimization were important predictors of paranoid ideation in adulthood.
7.	Hearing and speech impairment at age 4 and risk of later non-affective psychosis	Fors et al., 2013 [13]	A prospective study	N = 318	Well Baby Clinic (WBC) records.	Research findings support the hypothesis that psychosis has a developmental aspect with presentation of antecedent markers early in childhood, long before the onset of disorder. The findings also highlighted the fact that early hearing and speech impairments are risk indicators for later non-affective psychosis and possibly represent etiological clues and potentially modifiable risk factors.
8.	Effects of maltreatment and parental schizophrenia spectrum disorders on early childhood social-emotional functioning: a population record linkage study	Matheson et al., 2017 [23]	Cross-sectional study	N = 69,116	Social-emotional functioning (social competency, prosocial/helping behaviour, anxious/fearful behaviour; aggressive behaviour, and hyperactivity/inattention).	The study results showed that maltreatment, poor social competency, aggressive behaviour and hyperactivity/inattention were moderately associated. In contrast, the small associations were revealed between maltreatment and poor prosocial/helping and anxious/fearful behaviours.
9.	Attenuated psychotic symptoms in children and adolescent offspring of patients with schizophrenia	Noguera et al., 2018 [29]	Cross-sectional study	N = 148	Prodromal Symptoms Scale (SOPS)	The results showed the significant mean differences of SZ-off on Attenuated Psychotic Symptoms (APS) and Community Control group (CC-off) on psychopathological scales.
10.	Childhood developmental abnormalities in schizophrenia: evidence from high-risk studies	Niemi et al., 2003 [16]	Review study	16 High Risk Studies	The articles reviewed here were identified by Medline searches using the key words ‘‘schizophrenia” and ‘‘high-risk” for papers published from January 1966 until February 2001, and from the bibliographies of the publications thus obtained.	Results of reviewed articles showed the several factors that predicts schizophrenia including; problems in motor and neurological development, deficits in attention and verbal short-term memory, poor social competence, positive formal thought disorder-like symptoms, higher scores on psychosis-related scales in the MMPI, and severe instability of early rearing environment.
11.	Comparison of behavioral problems between the children with one schizophrenic parent and children with healthy parents	Sadeghi et al., 2020 [30]	Descriptive study	N = 120	The Child Behavior Checklist (CBCL)	The research results showed that the behavioral problem of primary school children with one schizophrenic parent was higher than children with healthy parents. However, there was no difference was observed concerning the behavioral problems among teenage participants.
12.	People With Psychosis Improve Affective Social Cognition and Self-Care After a Mindfulness-Based Social Cognition Training Program (SocialMIND)	Mediavilla et al., 2021 [31]	Intervention study	N = 38	1. Social cognition (Eyes Test). 2. Ambiguous Intentions and Hostility Questionnaire [AIHQ]. 3. Hinting Task. r. Social functioning tasks (Personal and Social Performance [PSP] scale)	The research findings revealed the significant improvement in social cognition and social functioning of psychosis outpatients after taking a SocialMIND Training Program.
13.	Short-term Social Skills Training in Schizophrenia Spectrum Disorders: A Clinical Trial in an Outpatient Setting	Takaloo et al., 2020 [24]	Intervention Study	N = 29	1. Global Assessment of Functioning Scale. 2. Positive and Negative Syndrome Scale. 3. World Health Organization Quality of Life Assessment (WHOQOL-BREF).	The study results suggested that social skills training play a significant role in reducing the positive and negative symptoms. Also, it improves the quality of life of Schizophrenic outpatients.
14	Recent Advances in Social Skills Training for Schizophrenia	Kopelowicz et al., 2006 [27]	Intervention Study	-	1. Techniques for Training Social Skills. 2. Evaluation of Social Skills Training. 3. Integrated Psychological Therapy. 4. Cognitive Enhancement Therapy	Social skills’ training is beneficial for schizophrenic patients in terms of acquiring interpersonal disease management and improving learning and behavioral functions.

*Note*. MK and RB extracted the data from the selected full-texts in December, 2023.

### Study selection

**Fig 1:**
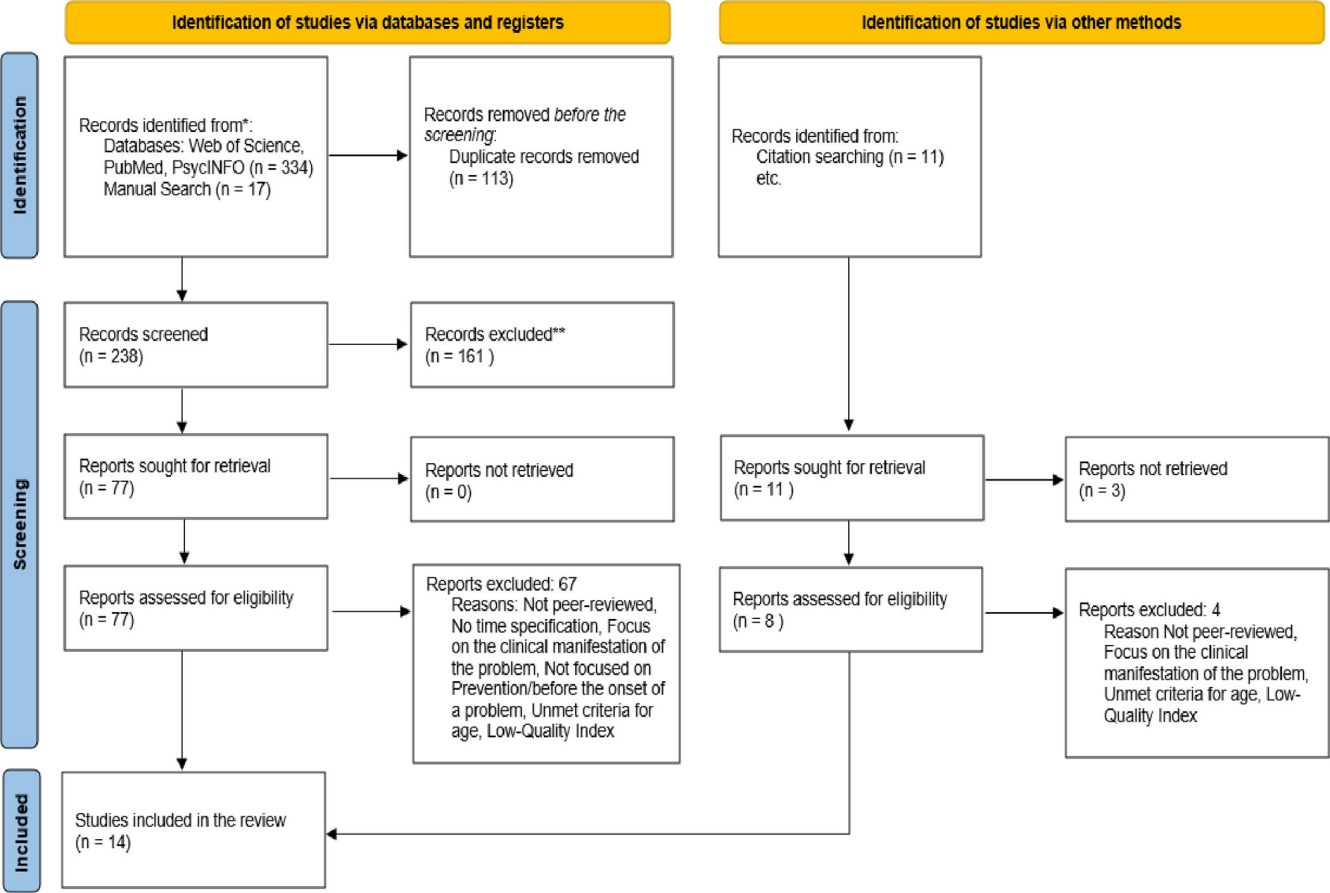
PRISMA diagram of the study selection and screening process.

We identified 351 articles through electronic and manual searches, which became 238 after removing duplicates. The title and abstract screening phase resulted in 77 full-text papers for review with the exclusion of 161 records. In the next step, we removed 68 full-text papers from the records of database searches and 4 out of manual search results. At the end of this whole process of screening and study selection (Fig 1), we found 14 studies for the qualitative synthesis of the findings concerning the objectives of our exploration.

### Description of the included studies

Our systematic review incorporated 14 studies on potential developmental markers of vulnerability to schizophrenia for those having a parental history of the disorder. The studies were published between 2003 to 2022, with a diversified sample of children and adolescents, conducted in nine countries. Table 1 represents the summary and other relevant details of the studies. Three Denmark, Two Iran, Two Spain, 2 Finland 1 Portugal, 1 Netherlands, 1 Sweden, 1 Australia, and 1 USA. The Quality index assessment presented six studies as good quality and the rest eight were rated as of fair quality.

## Results

**Fig 2:**
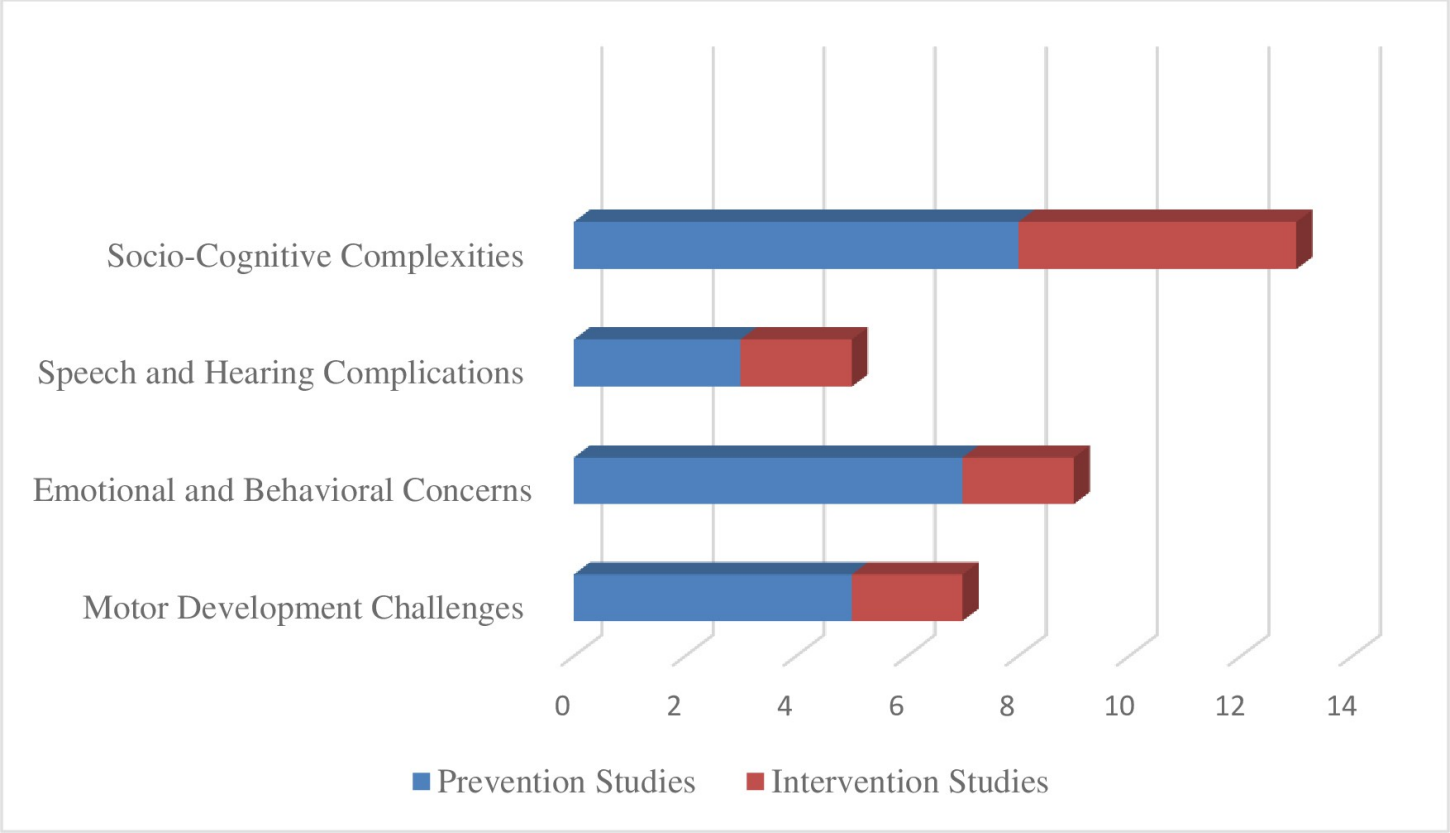
MESS typology for growing with schizophrenic parental history.

Fourteen articles were included in this systematic review, with a total of over 137,484 participants. We identified four domains (Fig 2) of functioning to be repeatedly highlighted in the studies as the potential contributors to the development of the problem. We seek to present them as the prevention zones and developmental checkpoints for rearing a child with a genetic predisposition to schizophrenia.

### Motor development challenges

We have identified four studies out of 14 highlighting that rearing a child with a parental history of schizophrenia requires careful consideration of motor development in the early years. Scientists tracked the developmental patterns of the birth cohort of 1966 and in their longitudinal findings, they elaborated that 1.5% of those who have a parental history of schizophrenia developed the problem till their 46 years of age [11]. It was elaborated that delays in holding the head up, grabbing an object, and walking with or without support were the prominent signals in the motor development of those individuals [19]. The statistical evidence also supported that motor delays are associated with the development of the problem later in life [20]. Researchers also addressed it as an outcome of parental schizophrenia interacting with birth complications or early developmental challenges resulting in the onset of the disorder in such children [21]. Therefore, this is one of the significant checkmarks needing careful consideration when a child is developing normally.

### Emotional and behavioral concerns

We identified six studies primarily focusing on emotional and behavioral challenges children with a parental history of schizophrenia experience which impair their functioning to the extent that they develop the disorder later on. Research presented the emotional disruptions, traumatic childhood like parental separation or death, apathy, and associated tendencies found very early among children [7]. It was further demonstrated that parental apathy, submissiveness, and bullying victimization of the child results in a variety of emotional issues and behavioral problems in children and this pattern later on contributes to the appearance of the disorder [22]. Such children were also found to be aggressive and withdrawn in social interactions worsening the behavioral adaptation on the whole [23]. Similarly, researchers pointed out that the positive and negative symptoms of schizophrenia are evident in the form of emotional and behavioral discrepancies genetically predisposed children exhibit in the early years of development [24].

### Speech and hearing complications

Three studies adamantly documented the speech or hearing complications being evident in the developmental history of children who develop schizophrenia later in life but had a parental history of the problem. It was highlighted that early speech and hearing impairment plays a risk factor in the development of the problem in children with a parental history of schizophrenia [13]. The Minnesota HR Study reported schizophrenic HR children performed poorly while responding to the tasks requiring sustained attention to discriminate signals or noise stimuli in different version of a tests covering continues performance aspects. Some other findings complemented the stance as interrupted speech, longer pauses while speaking, pitch variability issues [25], and instability of early rearing environment results in neurological deficits, verbal memory issues, speech, and hearing challenges documented as the points to be considered cautiously during the development of a child having a parental history of the problem [16] to prevent its development.

### Socio-cognitive complexities

We identified the last and potentially serious aspect being shadowed under various factors during the development of a child is the Socio-cognitive development of a child. Most of the studies in general and seven out of the total, in particular, targeted this area to be consciously and thoroughly monitored for the normal functioning of the child associated with the preventive measures when the genetic/familial history of the disorder is present. Researchers presented the social and emotional issues that children exhibit in the early developmental period [26]. The Copenhagen HR Study found that schizophrenic HR children gave more idiosyncratic and fragmented responses in the completion of word association task. Whereas, in Stony Brook HR Study such children reflected more of cognitive slippage or poor control of thoughts and their inappropriate verbal expression even at school-age and later in adolescence. Further, socio-economic status, familial background, or parental education were also considered as the social or environmental challenges for the child [21]. Also, executive impairments in the development of 7-year-old children [22] and marked social dysfunctioning [23].

### Skills training

The timely identification of a particular issue as a developmental trouble for the child, the probable functioning can be managed with tailored assistance. We found that social skills training [24], social-cognitive training [26], and need-based social competence development training [27]. All these activities consider the need for the person exhibiting the MESS symptomology to be delivered before worsening the specific problem.

## Discussion

We designed a systematic review of the developmental pattern of healthy children having a parental history of schizophrenia developing the disorder later in life. The focus is on tracking the developmental anomalies associated with the onset of the disorder to be tackled timely preventing the development of the disorder. We also tried to incorporate the available intervention training to be introduced when required instead of ignoring the existence of the preliminary aspects of the problem. Our findings present a detailed explanation of four domains requiring serious attention during the development of an individual with such a familial history. MESS typology incorporates motor developmental problems, emotional and behavioral issues, speech/hearing complications, and socio-cognitive dysfunctioning.

Previous studies present the delay of motor developmental milestones among children who later develop schizophrenia. The children exhibit trouble in standing up, grabbing objects, and walking without support. The findings were further supported in comparison to the healthy children and the situation was stronger in those having a parental history of the problem [20]. Further, one needs to be careful about the neurological functioning of the child, as neurological deficits, verbal memory issues; and speech, and hearing challenges are also documented to be massive challenges in the development of children with a parental history of the problem [17].

Moreover, it is presented that impairment in social functioning, social interactions, recognition of emotion, and problem-solving are indicators of a child’s vulnerability to developing schizophrenia later in life [26]. As our focus is on parental history, so, these are the additional aspects to be carefully monitored in and timely addressed. Also, social skills include appropriate social functioning, taking the understanding of right time and right behavior into account. However, generally in the development of children, we never take such aspects seriously taking them as lessons to be learned with time and growth but there is a clear need not to ignore such points in the development of these children with a specific history of the problem [25].

Additionally, school performance, working memory deficits, verbal memory complications, adjustment, and adaptation to the surroundings are some other factors not to be missed while rearing such children [28]. A proper check on the child’s executive functioning, storytelling, role-playing, emotional detachment, submissiveness, and anything that has some potential for deviance needs to be carefully monitored until the independently substantial functioning of the children. A careful eye coupled with desired training or exposure can not only reduce but also diminish the chances of the development of the disorder [24].

### Limitations and suggestions

We have just incorporated the peer-reviewed articles from the three specified databases, a relatively limited pool of studies. Thus, one can look up more with some additions to our very specific focus for having a more evidence-based understanding of developing schizophrenia. Also, due to the fewer number of studies in our full-text papers, we feel the exact temporal specification of the symptoms was not possible. Further studies can make this subject clearer and more exact for the understanding of parents or caregivers from the common public who do not have easy relevance to the scientific knowledge or at times are not aware of the terminologies or specifications of the disciplines.

### Implications

Our simple and actual focus was to provide a guideline or a comprehensive summary of crucial checkpoints for the parents or caretakers of children having a parental history of schizophrenia. We have presented the key areas to track the development of the child and potential strategies to tackle the concerns at the very moment they appear in a somewhat simple and to-the-point manner to help the common people dealing with such a child. Our attempt provide specific directions for the need and efficacy of some longitudinal studies to covering the indicators of potential risk factors among the high risk individuals. This can further help scientists to consider the environmental risk factors associated with schizophrenia like neurological deviations or prenatal infections. Past research also suggested to conducted follow-up studies with adults to track the development of the problem. More such studies will probably bring more clarity for the preventive efforts. Also, this comprehensive account of the developmental patterns would be a guide for the practitioners, parents and caregivers to be aware of potential dangers and the type of functional disturbance during the development of a child.

The practitioners, well-being centers, and skills trainers can also take advantage of scientific documentation of the problem to plan their strategies. Lastly, this review directs the gaps like temporal preferences, physiological or neurological advancements, and lack of governmental policies regarding such serious issues to be addressed both in research and practice.

## Conclusion

We presented a MESS typology to find the developmental checkpoints essential to be carefully monitored in the development of a child with a parental history of schizophrenia. This review serves two purposes, guiding how to be careful in rearing a child with a predisposition to the problem to preventing the onset of it and providing a direction on how to timely tackle the issue. Again, an attempt is directed to not only reduce but also prevent the occurrence of the disorder. Also, this would reduce the burden of lack of awareness and the constant stress parents and caregivers are experiencing about ‘what are the aspects to be taken care of’ while rearing such a child.

## Supporting information

S1 Checklist PRISMA 2020 for abstracts checklist. (DOCX)

S2 Checklist PRISMA 2020 checklist. (DOCX)
